# Pericytes require physiological oxygen tension to maintain phenotypic fidelity

**DOI:** 10.1038/s41598-024-80682-x

**Published:** 2024-11-28

**Authors:** Tamara McErlain, Elizabeth C. McCulla, Morgan J. Glass, Lauren E. Ziemer, Cristina M. Branco, Meera Murgai

**Affiliations:** 1 Laboratory of Cancer Biology and Genetics, National Cancer Institute, National Institutes of Health, Bethesda, MD UK; 2 Patrick G. Johnston Centre for Cancer Research, Queen’s University Belfast, Belfast, UK

**Keywords:** Pericytes, KLF4, Metastasis, Hyperoxia, Physioxia, Lineage plasticity, Breast cancer, Cancer microenvironment, Metastasis, Angiogenesis, Cardiovascular models, Cancer models, Cell culture

## Abstract

Pericytes function to maintain tissue homeostasis by regulating capillary blood flow and maintaining endothelial barrier function. Pericyte dysfunction is associated with various pathologies and has recently been found to aid cancer progression. Despite having critical functions in health and disease, pericytes remain an understudied population due to a lack of model systems which accurately reflect in vivo biology. In this study we developed a protocol to isolate and culture murine lung, brain, bone, and liver pericytes, that maintains their known phenotypes and functions. We demonstrate that pericytes, being inherently plastic, benefit from controlled oxygen tension culture conditions, aiding their expansion ex vivo. Primary pericytes grown in physiologically relevant oxygen tensions (10% O_2_ for lung; 5% O_2_ for brain, bone, and liver) also better retain pericyte phenotypes indicated by stable expression of characteristic transcriptional and protein markers. In functional tube formation assays, pericytes were observed to significantly associate with endothelial junctions. Importantly, we identified growth conditions that limit expression of the plasticity factor *Klf4* to prevent spontaneous phenotypic switching in vitro. Additionally, we were able to induce pathological pericyte phenotypic switching in response to metastatic stimuli to accurately recapitulate in vivo biology. Here, we present a robust method for studying pericyte biology in both physiology and disease.

## Introduction

Pericytes are critical components of microvascular homeostasis and are found across heterogeneous capillary beds^1,2^. Pericyte detachment or loss is associated with microvascular dysfunction observed in many pathologies, including aging, ischemic disease, neurodegeneration, and cancer^3^. Pericyte pathologies also extend beyond vascular dysfunction^4^, involving extracellular matrix (ECM) remodeling and fibrosis^5,6^. We and others have previously found that pericytes are critical to promoting cancer metastasis^7,8^.

Despite growing clinical interest in pericytes for their role in disease and regenerative medicine, the field has struggled to develop an in vitro toolkit for pericyte research that is reflective of physiology, relatively cost-effective, and high throughput^3^. This is in part due to the heterogeneity of origin^9,10^, function^2^, and molecular markers^1,11^, which has made it difficult not only to define pericytes but also to model them in a physiologically accurate way. The inherent phenotypic plasticity of pericytes, which allows them to differentiate into fat, muscle, cartilage, and bone cells^12–14^ may make them particularly sensitive to environmental cues. This sensitivity may in fact underly the inconsistences observed between pericyte isolation and culturing methods which in turn inevitably results in discrepancies in data obtained from different protocols, clouding reliability and translational value of the associated findings. Here, we sought to identify an in vitro pericyte modeling system that would most faithfully represent pericyte homeostatic and pathological phenotypes.

The optimal pericyte model should support expansion of cells for high throughput studies while accurately recapitulating pericyte biology without inducing spontaneous differentiation or pathological activation. The differentiation potential of pericytes presents a challenge for establishing reproducible cell culture models; however, similar difficulties have been overcome in the culture of pluripotent stem cells by regulation of oxygen tension^15–18^. In vivo, different tissues experience varying oxygen levels: while ambient atmospheric oxygen is 150 mm/Hg (equivalent to 21% O_2_), arterial blood is closer to 100 mm/Hg, or 14% O_2_^19^_._ The O_2_ tension in capillary beds is dependent on the rate of capillary blood flow, density of the capillary bed, interstitial fluid composition, and pressure, which varies from organ-to-organ, and by vessel morphology^20^. Lung pericytes are found at the interface of alveolar capillaries where they are in intimate connection with the lung endothelium. This compartment is exposed to oxygen tensions of 75–100 mm/Hg, or 10–13% O_2_^21,22^, while brain, bone, and liver capillary beds typically encounter 1–5% O_2_^23–30^. Standard tissue culture practice maintains cells at ~ 21% O_2_; however, mimicking in vivo oxygen tension can more accurately reflect cell identity, function, and response to stimuli^23,24^. Physiological oxygen tension has been demonstrated to be an important factor of stem cell niches, functioning in cell fate determination with low oxygen tension being essential to maintain the plasticity and proliferation of stem cells^15–18^. Importantly it has been demonstrated that hyperoxia exposure impacts many cellular processes and limits the ability of in vitro tools to replicate in vivo biology^31,32^.

Pericytes have a functional role as oxygen and metabolic sensors to meet local tissue nutrient demands^33,34^. This, combined with their inherent plasticity, might suggest that this population is particularly sensitive to oxygen tensions and downstream metabolic pathways. Using the knowledge available in the microvascular field^35,36^, we developed a protocol for the isolation of murine pericytes for in vitro studies. We investigated if pericytes require physiological oxygen tension to maintain their homeostatic phenotype and response to pathological cues. Learning from the culturing practices of other pluripotent populations, in this study we sought to evaluate the understudied role of physiological oxygen tension to better model and retain pericyte phenotype and function. We report the development and characterization of a model that captures the phenotypic plasticity of pericytes observed in vascular pathologies, including cancer.

## Results

### Physiological oxygen levels support the growth of primary lung pericytes ex vivo

**Fig. 1:**
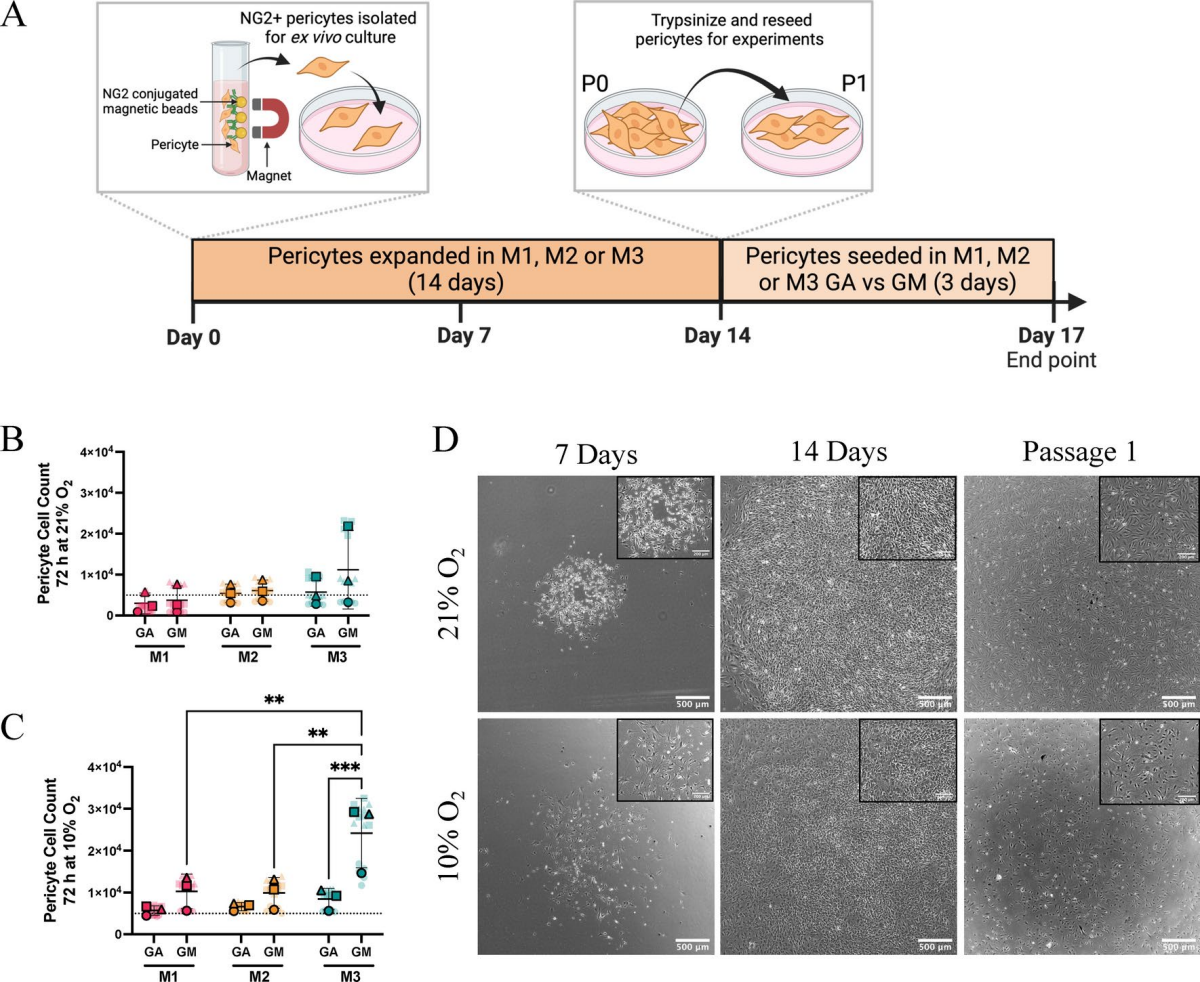
Identifying culture condition that support primary lung pericyte expansion ex vivo. (**A**) Schematic demonstrating the timeline from pericyte isolation to use in experiments. (**B–C**) Pericyte proliferation over 72 h in response to both the growth arrest (GA) and growth medium (GM) compositions of the media to be optimised (M1-3). Proliferation at 21% O_2_ is displayed in (**B**) and 10% O_2_ in (**C**). Dotted line represents seeding density. Statistical significance was assessed by two-way ANOVA with Tukey’s multiple comparisons test (**B–C**) (**p < 0.01, **p <  0.001). Data are displayed as Superplots (Large symbols: means of experimental replicates; Small symbols: technical replicates for each experimental replicate; Lines: mean ± SD of replicate means. Statistical analysis was performed on the mean experimental replicate data only. (**D**) Representative phase contrast images of primary lung pericytes expanded in medium composition M3 and maintained in 21% O_2_ or 10% O_2_. Key time points during the expansion of primary pericytes are displayed. Schematic made in Biorender.com.

To examine pericyte responses to oxygen levels in vitro, NG2-expressing pericytes isolated from mouse lungs were expanded in 21% O_2_ or 10% O_2_ for 14 days (passage 0), after which they were trypsinized and reseeded (passage 1) to assess their proliferative potential (Fig. 1A). Phase contrast images were obtained at key time points during the expansion of pericytes, and proliferation was assessed at passage 1 (Fig. 1A). To identify media conditions that support cell growth while maintaining pericyte biology, we developed a growth medium (GM) to promote pericyte culture expansion and a growth arrest (GA) medium to model the proliferative quiescence of pericytes in vivo. Three media compositions (Supplementary Table 1) were tested at both 21% O_2_ (Fig. 1B) and 10% O_2_ (Fig. 1C) for the ability to induce pericyte proliferation or to maintain proliferative quiescence over 72 h. Medium 1 (M1) was a commercially available pericyte growth medium, medium 2 (M2) was an endothelial cell growth medium without additional growth factors, and medium 3 (M3) was the same endothelial cell growth medium supplemented with endothelial cell growth factors. The GA version of each medium composition had reduced serum and no additional growth factors. GM composition M3 supported pericyte culture expansion better than M1 and M2 under 10% O_2_. Additionally, the GA version of composition M3 effectively limited pericyte proliferation over the seeding density (indicated by the dashed line) in both oxygen tensions, maintaining pericytes in proliferative quiescence. The ability to effectively reduce proliferation accurately models pericyte quiescence associated with blood vessel investment under homeostatic conditions.

Phase contrast images were taken during the course of pericyte expansion to assess morphology in each medium. At 7 d post isolation, cells in both 10% and 21% O_2_ had visible pericyte colonies. However, only M3 achieved confluence by 14 d (Fig. 1D), with M1 and M2 remaining as small colonies (Supplemental Fig. 1). Pericytes maintained in M1 or M2 exhibited morphological signs of senescence after passage 1 in 10% O_2_ (Supplemental Fig. 1). In contrast, pericytes from M3 maintained in 10% O_2_ retained typical pericyte morphology with multiple long cellular projections after passage 1. However, pericytes maintained in 21% O_2_ appeared fibroblastic, indicated by the spindle-shaped cell morphology and parallel alignment^37^ (Fig. 1D). These data suggested that the oxygen tension under which pericytes are grown can influence phenotypic status, indicated by differences in cell morphology.

### Primary lung pericytes cultured in 10% O_2_ demonstrate stable transcript expression of characteristic genes

**Fig. 2:**
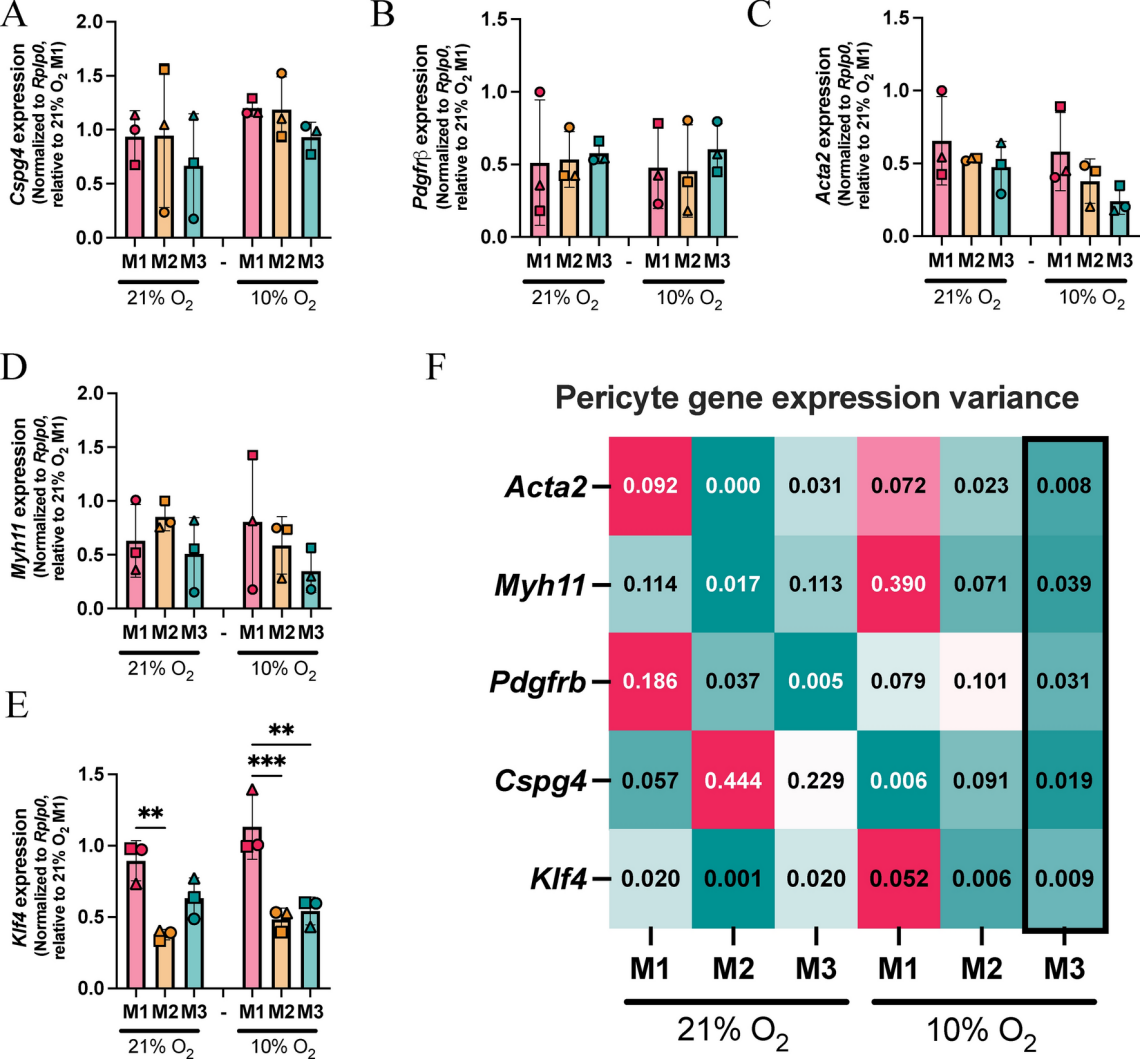
10% O_2_ reduces gene expression variation between lung pericyte batches. (**A**) RT-qPCR was performed on pericytes expanded in three different medium compositions under 10% O_2_ and 21% O_2_ to assess transcriptional expression of the characteristic pericyte genes *Cspg4* (**A**), *Pdgfrb* (**B**), *Acta2* (**C**), *Myh11* (**D**) and *Klf4* (**E**). Data are displayed as average fold change per experimental replicate ± SD (N = 3). Statistical significance was assessed by one-way ANOVA with Tukey’s multiple comparisons test (*p < 0.05, **p < 0.01). Variance of pericyte gene expression data is plotted in the heat map (**F**).

To assess the culture conditions that best-retained pericyte identity ex vivo, we analyzed changes in expression of *Cspg4* (Fig. 2A) and *Pdgfrb* (Fig. 2B), pericyte identification markers, under physiologically relevant GA conditions with 10% O_2_ compared to 21% O_2_. Interestingly, expression of both genes was retained in all experimental conditions. The majority of NG2 + pericytes are reported to express contractile genes such as *Acta2* and *Myh11*^38,39^. We therefore evaluated *Acta2* (Fig. 2C) and *Myh11* (Fig. 2D) gene expression and found that they were also retained ex vivo in all conditions. Variance in gene expression was calculated between independent pericyte batches and across culture conditions, demonstrating that composition M3 and 10% O_2_ promoted stability of transcriptional expression (Fig. 2F).

It is reported in pathologies, including cancer, that pericytes undergo phenotypic switching, indicated by increased expression of the pluripotency gene, *Klf4*^8,40,41^*.* Pericyte *Klf4* expression was observed to be significantly increased when maintained in medium composition M1 under both oxygen tensions (Fig. 2E and F) suggesting that it had an ‘activating’ effect on pericytes. Here we have identified the culturing conditions that retain characteristic pericyte identification and functional genes, while minimising the expression of the activating factor *Klf4*, to model physiologically relevant pericyte biology. The data suggest that medium composition M3 will aid reproducibility of experiments by limiting the gene expression variation between independent pericyte batches.

### Physiologically relevant oxygen tensions aid culture of pericytes from multiple organ sources

**Fig. 3:**
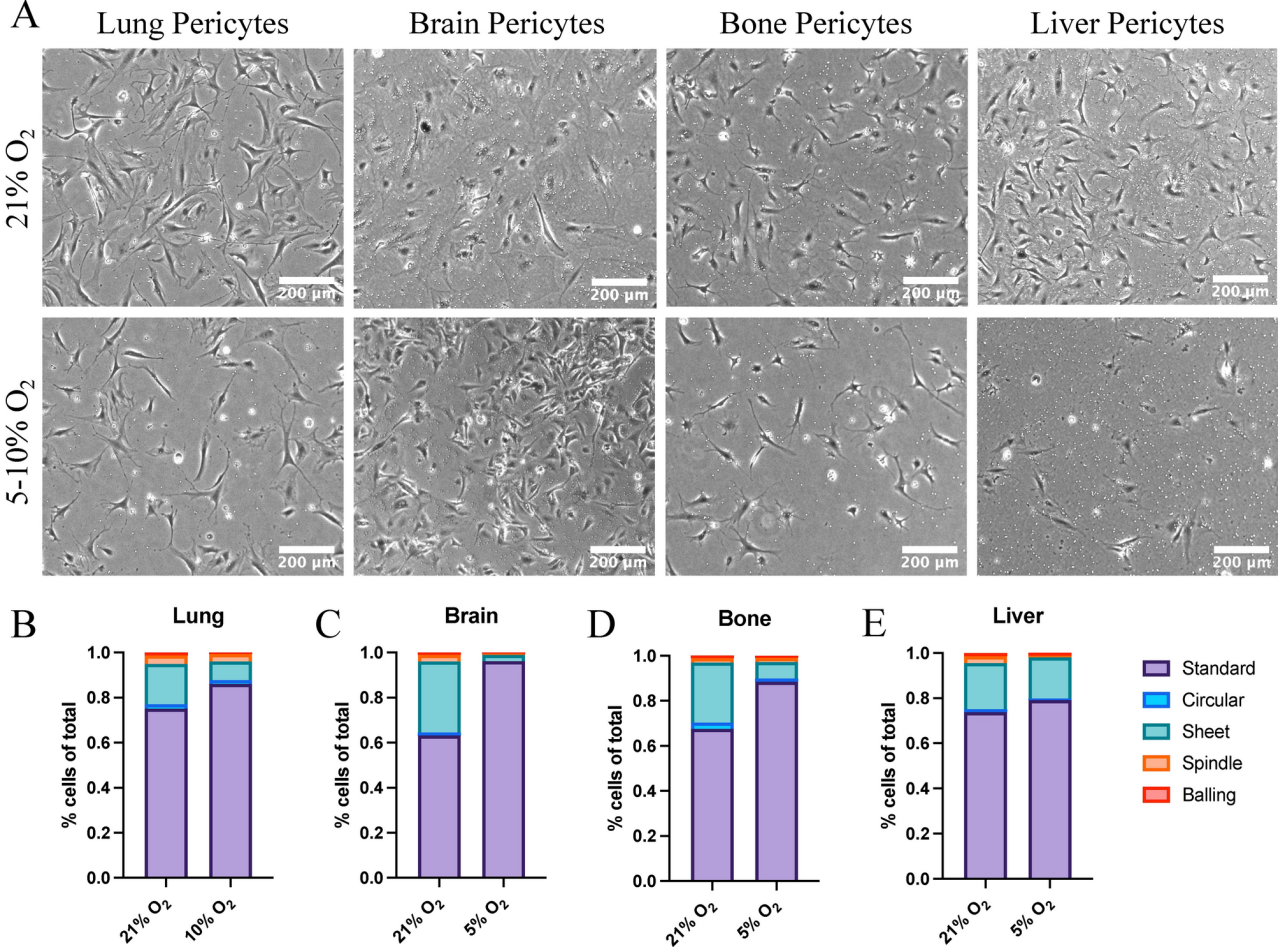
Oxygen tension impacts primary pericyte morphological heterogeneity. (**A**) Representative phase contrast images of primary lung, brain, bone, and liver pericytes expanded in M3 and maintained in either 21% O_2_ or lungs at 10% O_2_ and brain, bone and liver at 5% O_2_. Scale bar = 200 mm. Pericytes were assigned a morphological classification and the proportions of each classification were compared between the specific oxygen tensions for lung, (**B**), brain (**C**), bone (**D**) and liver (**E**).

Pericytes are found throughout the body and are highly heterogenous in function and morphology based on their location along capillary beds as well as between different organs^11,42,43^.To test whether our protocol would be applicable to pericytes derived from multiple organ sites beyond the lung, NG2-expressing cells were isolated from brain, bone, and liver. In vitro cultures resembled typical pericyte morphology with a prominent cell body and multiple long extensions regardless of anatomic origin (Fig. 3A, Supplemental Fig. 2). Using an established protocol for quantifying pericyte morphological phenotypes in vitro^44^, pericyte morphology was compared between 21% O_2_ and physiologically relevant oxygen tensions for each organ-derived cell culture (5% O_2_ for brain, bone, and liver; 10% O_2_ for lung). Standard morphology pericytes were enriched in pericytes cultured in physiologically relevant oxygen tensions compared to those cultured at 21% O_2_ (Fig. 3B–E). Previous work has demonstrated that Standard morphology pericytes are enriched in early passage human brain vascular pericytes, which decreases at later passages^44^.

**Fig. 4:**
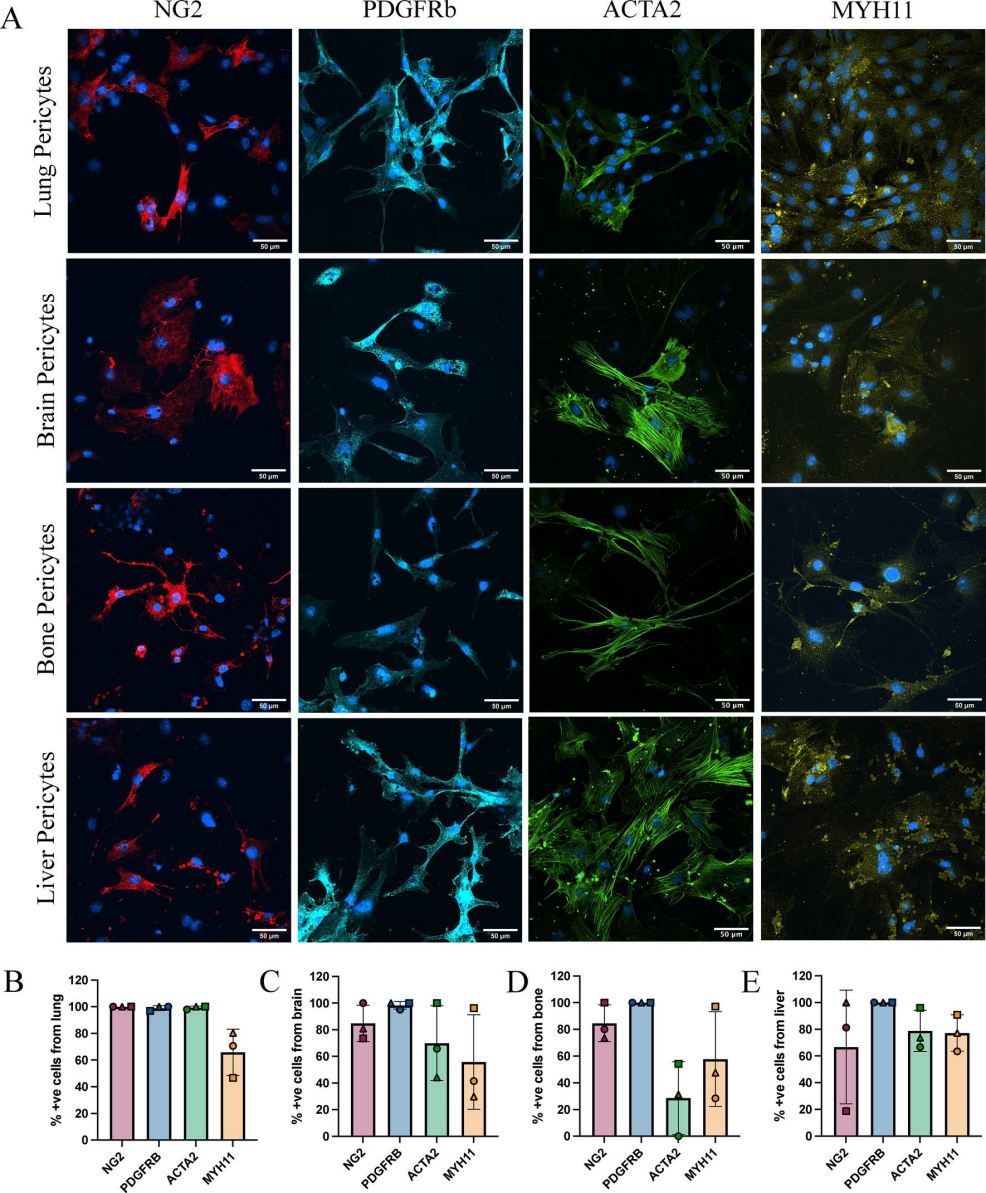
Immunofluorescence characterization of primary pericyte cultures from mouse. Pericytes obtained from mouse lung, brain, bone, and liver were seeded at passage 1 for immunofluorescence study (**A**) A panel of pericyte identification markers including NG2, MYH11, PDGFRb, and ACTA2 were used to characterize pericyte marker expression in different organs. One representative image is displayed from one pericyte batch. Scale Bar = 50 mm. The percentage of cells positive for each of the markers was quantified from three independent batches of cells isolated from the lung (**B**), brain (**C**), bone (**D**) and liver (**E**).

Protein expression of characteristic pericyte markers was validated across all organs using immunofluorescence for 4 key proteins, NG2, PDGFRb MYH11, and ACTA2 (Fig. 4A). 80–100% of the cells were positive for the pericyte markers NG2 and PDGFRb, suggesting a high degree of overlap for these markers within pericyte cultures from all organs (Fig. 4B–E). A heterogeneous staining pattern was observed for ACTA2 and MYH11 within the pericyte cultures, suggesting this culture method can capture the heterogeneity of pericyte phenotypes observed in vivo along the microvascular tree, whereby contractile protein expression varies across pericyte phenotypes. Capturing multiple pericyte subtypes provides a more accurate representation of the in vivo pericyte population, which will improve the translational relevance of this model system.

### Ex vivo pericyte cultures demonstrate a functionally relevant association with endothelial junctions

**Fig. 5:**
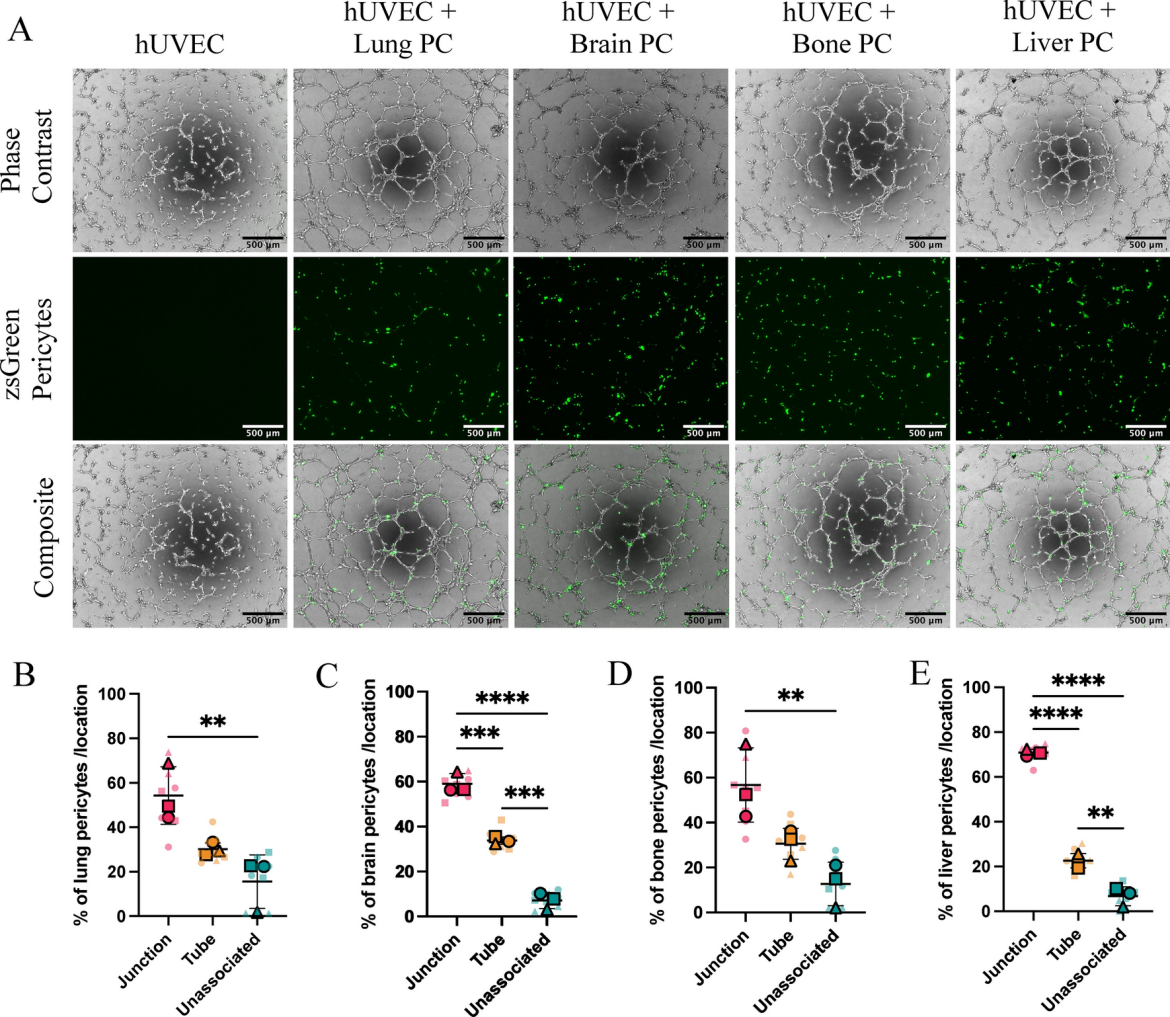
Ex vivo pericyte retain physiological function at endothelial junctions. hUVECs (unlabeled) were co-cultured with pericytes (zsGreen) at a ratio of 1:10 (lung, bone, and liver) or 1:5 (brain) PC:EC in Matrigel over 4 h. Representative images from 4 h co-culture are displayed (**A**). Scale Bar = 500 µm. Quantification of pericyte association with endothelial tubes is quantified as the number of zsGreen positive cells found at junctions, tubes, or unassociated (**B–E**). Statistical significance was assessed by one-way ANOVA with Tukey’s multiple comparison test (**p < 0.01, ***p < 0.001, ****p < 0.0001). Data are displayed as Superplots (Large symbols: means of experimental replicates; Small symbols: technical replicates for each experimental replicate; Lines: mean ± SD of replicate means). Statistical analysis was performed on the mean experimental replicate data only.

A key physiological role of pericytes is to support blood vessel formation and structural integrity. To determine if pericytes retained their physiological function in vascular support ex vivo, tube formation assays were performed using human umbilical vein endothelial cells (hUVECs) in co-culture with pericytes from NG2-ERT^2^-cre Rosa-STOP-flox-zsGreen mice. Pericyte density on vessels is known to correlate with the barrier function unique to each organ^45^. Pericyte density is highest in the central nervous system (CNS) with up to 70–80% vessel coverage^46,47^ compared to the lungs and liver, having an approximately 1:10 ratio of pericytes to endothelial cells (ECs)^45^. To account for these differences in pericyte investment, tube formations were performed with pericytes from lung, bone, and liver co-cultures used at a 1:10 ratio while brain pericytes were co-cultured at a 1:5 ratio of pericyte to ECs. hUVECs demonstrated alignment and adhesion in Matrigel to form tube-like structures. Pericytes isolated from the lung, brain, bone, and liver when added to co-culture associated with the endothelial tubes. Representative images are shown for the 4 h time point in each culture condition (Fig. 5A). The tube formation images were analyzed to determine the localization of pericytes to endothelial tubes and were assigned junction, tube, or unassociated (Supplemental Fig. 3A). Pericytes from all organs were significantly associated with endothelial junctions (Fig. 5B–E), however the presence of pericytes had no significant impact on total hUVEC mesh area compared to hUVECs alone (Supplemental Fig. 3B–E). These data support known pericyte physiological function and location on branch points in the vasculature^48^, indicating that these pericyte cultures retain physiologically relevant functions.

### Primary lung pericytes cultured at 10% O_2_ accurately model tumor-derived factor induced activation

**Fig. 6:**
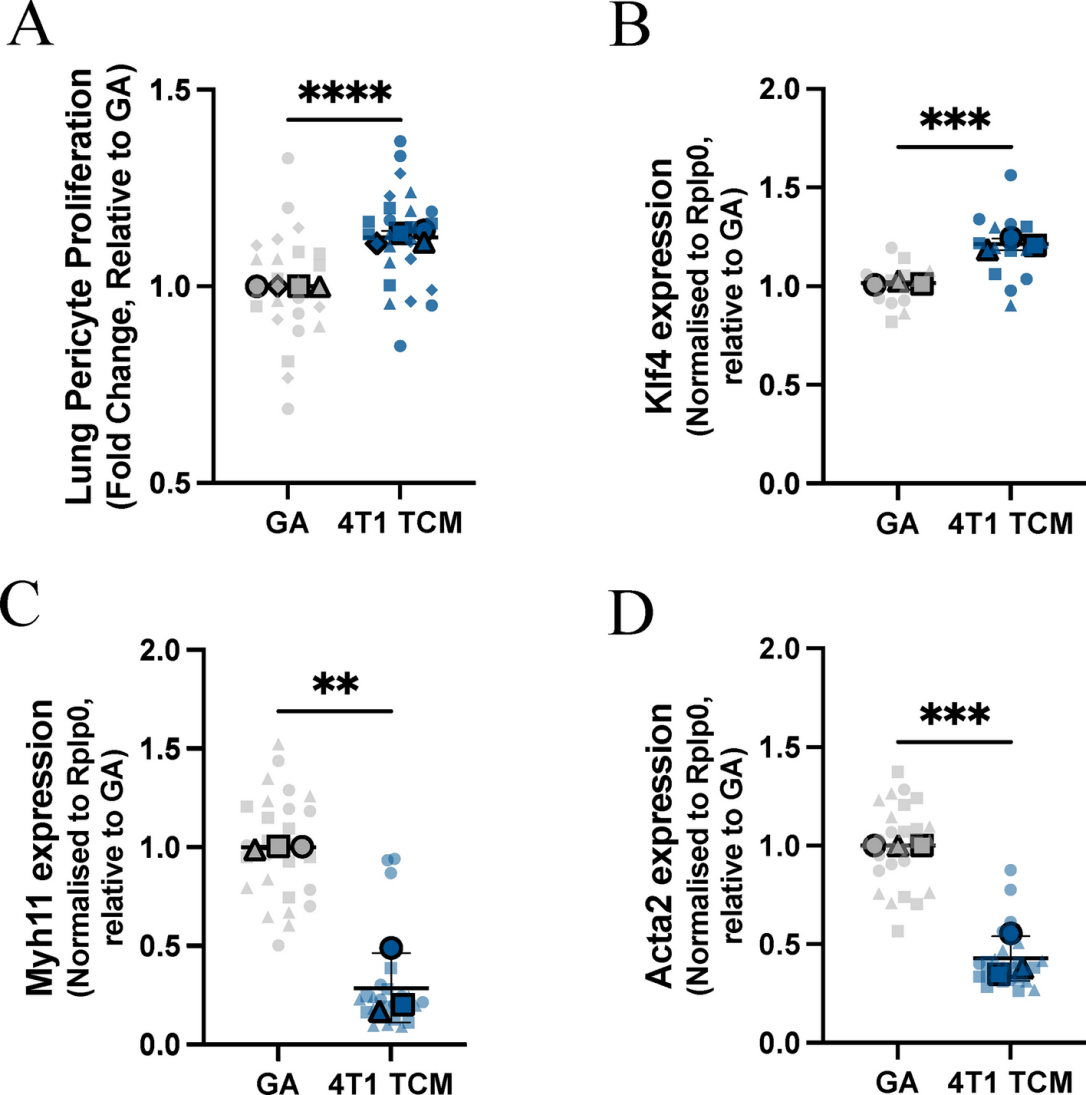
Ex vivo pericyte cultures accurately recapitulate in vivo phenotypic switching. (**A**) Pericyte phenotypic switching ex vivo was assessed by treating lung pericytes with TCM from the metastatic breast cancer cell line 4T1. Lung pericyte proliferation in response to TCM was assessed at 72 h (**A**) and *Klf4* expression was assessed by RT-qPCR at 30 min following TCM treatment (**B**). Expression of characteristic perivascular marker genes, *Myh11* (**C**) and *Acta2* (**D**) were assessed after 72 h of TCM treatment compared to the GA control. Statistical significance was assessed by unpaired t tests (**p < 0.01, ***p < 0.001, ****p < 0.0001). Data are displayed as Superplots (Large symbols: means of experimental replicates; Small symbols: technical replicates for each experimental replicate; Lines: mean ± SD of replicate means). Statistical analysis was performed on the mean experimental replicate data only.

Our previous work has demonstrated that metastatic tumor-derived factors promote pericyte activation, characterized by pericyte proliferation, down-regulation of perivascular marker genes, and an increase in *Klf4* expression in vivo^8^. Pericyte phenotypic switching has also been observed in diabetic retinopathy and acute tissue injury^40,41^. To examine whether ex vivo pericyte cultures recapitulate our previous in vivo findings, lung pericytes were treated with tumor cell conditioned medium (TCM) collected from the metastatic triple-negative breast cancer cell line, 4T1. Pericyte activation conditions were pre-determined by assessing proliferation in response to different concentrations of TCM and monitoring *Klf4* expression over time (Supplemental Fig. 4). Pericytes were treated with 50% TCM for 72 h, and proliferation was assessed compared to the GA control. 4T1 TCM induced significant pericyte proliferation (Fig. 6A). The expression of the pluripotency factor, *Klf4*, was also significantly increased in pericytes 30 min after exposure to 4T1 TCM compared to the GA control (Fig. 6B). Additionally, perivascular cell marker genes, *Myh11* and *Acta2* were significantly downregulated in pericytes treated with 4T1 TCM after 72 h compared to GA (Fig. 6C,D). These parameters together indicate that 4T1 TCM induced pericyte phenotypic switching like that observed in vivo*,* suggesting that these pericyte cultures can be used to study the role of pericytes in metastasis.

## Discussion

Historically, it has been difficult to model pericyte biology in vitro due to low yield and lack of standardized methodologies^3^. Limited pericyte cell lines are available and major problems are associated with the current tools. Under homeostatic conditions pericytes are quiescent and not actively proliferating unless required in angiogenesis or response to injury^49^ whereas immortalised cell lines are induced to permanently proliferate^50^, a phenotype associated with perivascular cell activation^8,51^. Pericytes have been generated successfully from induced pluripotent stem cells (iPSC)^52–54^ however iPSC can be difficult to produce and require highly specific culturing conditions^55,56^. Primary cell lines are a useful tool to recapitulate in vivo variation and physiology but are limited by expansion capabilities and the retention of cellular function and phenotype ex vivo^3,57^. These challenges have resulted in pericytes remaining an understudied population despite their demonstratably vital functions in both physiology and disease.

In this study we present an NG2-specific antibody conjugated magnetic bead pull down protocol for the isolation of pericytes from multiple organ sources. We focused on NG2-expressing pericytes because we have previous found that NG2 + pericytes promote cancer metastasis^8^. Although NG2 is also expressed in oligodendrocyte progenitors (OPCs) in the brain we observed no evidence of OPCs after culture for 14 days, suggesting that pericyte-promoting in vitro conditions may select against this population^58^. Future work will be necessary to profile the initial NG2-selected cell population, which is currently limited by the very low cell numbers that are recovered at this step. Additionally, NG2 appears to be expressed continuously on arteriole and mid-capillary pericytes and therefore should capture a range of known pericyte subtypes^59^; however, in addition or alternatively to NG2, pericytes can express proteins such as PDGFRb^3,60^. Future studies may highlight whether a similar approach could be used for alternative pericyte subpopulations including PDGFRb + NG2- pericytes, particularly along post-capillary venules where pericytes are thought to lack NG2 expression^59,61^.

Optimization of pericyte culturing conditions highlighted the importance of maintaining primary cultures under physiologically relevant oxygen, as oxygen tension is becoming increasingly appreciated as a mediator of cellular phenotypic switching^62,63^. For example, 5% O_2_ was found to be critical in maintaining hepatocyte morphology and function ex vivo, with cultures maintained at 21% O_2_ rapidly undergoing epithelial-mesenchymal transition (EMT) and losing hepatocyte functions including albumin production, low-density lipoprotein (LDL) uptake, and expression of drug metabolizing enzymes^32^. We have previously found that culturing microvascular endothelial cells (ECs) at 21% O_2_ impaired the cellular response to hypoxia, when compared to those cells that had been maintained in oxygen tensions that matched those found in their tissue of origin^31^. Consistent with these reports, pericyte maintenance in physiologically relevant oxygen tensions better recapitulated pericyte morphology, characteristic marker expression and association with endothelial tubes.

Pericytes exist as a continuum along microvascular arterioles to venules and have unique functional roles depending on location^1^, with ensheathing pericytes acting to rapidly control blood flow, and thin-strand pericytes functioning as metabolic sensors^34,64^. Expression of contractile proteins such as ACTA2 and MYH11 vary across these pericyte sub-types in vivo, and correlate with contractile function and morphology in vitro^11,42–44^. Additionally, each organ has a specialized microvascular network to carry out distinct functions. Continuous capillaries, which are found in most organs including the brain and retina, are barrier forming, whereas sinusoidal capillaries, found in the liver and bone marrow, are discontinuous and allow free movement of materials^65^. Recent studies have noted that pericytes analyzed from multiple organs retain expression of organ-specific solute carriers critical for their organ-specific function^66,67^. Importantly, we found that pericytes isolated from lung, brain, bone and liver displayed heterogeneity in morphology and expression of functional proteins that recapitulates in vivo observations, suggesting that this method preserves pericyte function and organ specificity. Consistent with their in vivo role in regulating vessel barrier function through paracrine signaling with endothelial cells^68,69^, we observed that ex vivo cultured pericytes associated closely with endothelial cell junctions in a co-culture model of endothelial tube formation. Future work will be needed to further delineate specialized in vitro pericyte behaviors, including in extracellular matrix remodeling capabilities and intercellular communication between pericytes, endothelial cells, and other components of the perivascular microenvironment.

An important objective when selecting in vitro pericyte culture systems is to faithfully model the in vivo features of pericyte function, with particular attention paid to the disease model being studied. The overall goal of our study was to create a pericyte culture model that could be used to better understand the role of pericytes in metastasis. Critical to the success of creating an in vitro primary pericyte model system was the ability to recapitulate lung pericyte activation during metastatic progression and other disease states, as was observed in vivo^8,40,41^. We found that primary lung pericytes expressed the stem/plasticity factor *Klf4* and increased proliferation in response to TCM from the metastatic breast cancer cell line, 4T1. Additionally, pericytes treated with 4T1 TCM also demonstrated reduced expression of characteristic perivascular marker genes *Myh11* and *Acta2*, suggesting that pathological phenotypic switching can be observed in thi*s *ex vivo system. These pericyte responses are hallmarks of their plasticity and activation during premetastatic niche formation^8^.

In this study, we have described a high throughput and reproducible pericyte cell culture system that is dependent on physiologically relevant oxygen tensions to accurately recapitulate pericyte biology. This tool will aid the study of pericytes in health and disease, including investigation into the organ specific roles of pericytes in metastasis.

## Materials and methods

### Animals

Pericyte cell cultures were obtained from female C57BL/6 mice or NG2-ERT^2^-cre Rosa-STOP-flox-zsGreen (NG2-zsGreen) mice at 12 weeks of age. Animals were anesthetized by isoflurane followed by cervical dislocation. All animals were maintained under pathogen free conditions within the NIH animal facility. To induce CreERT-mediated recombination in NG2-zsGreen mice, intraperitoneal injections of tamoxifen (T5648, Millipore Sigma) were administered. Tamoxifen was dissolved in corn oil (C8267, Millipore Sigma) to a final concentration of 10 mg/mL and 100 µL was injected once a day for 10 d total (5 d on, 2 d off, 5 d on) followed by a one week wash out period. All animal experiments were conducted in accordance with the guidelines and regulations outlined in the Guide for the Care and Use of Laboratory Animals and were approved by the NCI-Bethesda Animal Care and Use Committee. All procedures were in accordance with those outlined in the ARRIVE guidelines.

### Primary pericyte isolation and culture

A protocol for the isolation of murine pericytes was developed by adapting a previously published protocol for the isolation of microvascular ECs^31,35^. The pericyte isolation protocol has been described in detail in supplemental methods and materials (Supplemental Fig. 5). Pericyte yield and behavior may be altered by many factors including age, sex, or pathology, therefore, to standardize the protocol only 12-week-old female C57BL/6 mice were used for pericyte cultures. Based on established endothelial cell culture protocols^70,71^, pericytes were cultured on plates coated with 0.06 mg/mL collagen (C7661, Millipore Sigma) and maintained in a 37 °C humidity-controlled incubator at physiologically relevant oxygen tensions (10% O_2_ for lungs or 5% O_2_ for bone, brain, and liver, with 5% CO_2_) and used for experiments within 10–14 d. Pericytes were maintained in medium containing a 1:1 mix of low glucose Dulbecco’s Modified Eagle’s medium (DMEM) (D6046, Millipore Sigma) and Ham’s F12 nutrient mix (11765047, Thermo Fisher Scientific), supplemented with 1% MEM non-essential amino acids (11140050, Thermo Fisher Scientific), 2 mM sodium pyruvate (11360070, Thermo fisher Scientific), 20 mM Hepes (15630080, Thermo Fisher Scientific), 1X endothelial cell growth factors (390599, Bio-techne), 1% penicillin/streptomycin (15070063, Thermo Fisher Scientific) and 20% fetal bovine serum (900–208-500, GeminiBio). Before experiments pericytes were placed in GA medium containing reduced FBS (2%) and no endothelial cell growth factors for 24 h.

### Cell lines and culturing conditions

Human umbilical vein endothelial cells (hUVEC) were provided by Clare Waterman (NHLBI, Bethesda, USA) and cultured on plastic in a 37 °C humidity-controlled incubator with 5% CO_2_. medium containing a 1:1 mix of low glucose (1 g/L) DMEM and Ham’s F12 nutrient mix, supplemented with 1% MEM non-essential amino acids, 2 mM sodium pyruvate, 20 mM Hepes, 2% endothelial cell growth factors, 10 mg/mL heparin (H3149, Millipore Sigma), 1% penicillin/streptomycin and 10% fetal bovine serum.

The metastatic murine 4T1 tumor cell line was provided by Kent Hunter (National Cancer Institute, Bethesda, USA). The 4T1 cell line was maintained on 2D plastic culture flasks in a 37 °C humidity-controlled incubator with 5% CO_2_ in RPMI (11-875-119, Thermo Fisher Scientific) with 10% FBS, 1% penicillin/streptomycin.

### Tumor cell conditioned media collection

Tumor cells were plated at 1 × 10^5^ cells/mL in a T75 flask with pericyte GA medium and allowed to proliferate for 72 h. The tumor cell conditioned medium (TCM) was then collected and filtered through a 0.22 μm syringe filter (380111, Nest Scientific), aliquoted and stored at − 80 °C for downstream experiments. The TCM stock was diluted 1:1 in pericyte GA medium before use in experiments.

### Proliferation assay

Pericytes were seeded on collagen coated 96-well plates at 5 × 10^3^ cells per well for lung pericytes and 1 × 10^4^ cells per well for brain, bone, and liver pericytes in GA medium and returned to the appropriate incubator to adhere overnight. Pericytes were treated with control GA medium or TCM from 4T1 tumor cells that was diluted 1:1 in GA medium. The treated cells were returned to the appropriate incubator, 72 h later the cell nuclei were stained with 2 µM Hoechst (62249, Thermo Fisher Scientific) in PBS and the cells were counted using the Celigo image cytometer.

### Quantitative real-time PCR

For optimisation of pericyte growth conditions RNA was collected from pericytes seeded at 1 × 10^5^ in a 12-well plate using the RNeasy Mini Kit (74,104, Qiagen) after 24 h in the appropriate GA medium. For pericyte phenotypic switching experiments, pericytes were seeded in GA medium and allowed to adhere overnight, the following day the cells were treated with 4T1 TCM or GA medium for 72 h, and then isolated as above. RNA was quantified using a Nanodrop and 200 ng was used for reverse transcriptase cDNA synthesis using the iScript Reverse Transcriptase Supermix (1708841, BioRad). RT-qPCR was performed using cDNA diluted 1:30 in RNA free water, EvaGreen master mix (1725212, BioRad) and 10 µM primers.

Alternatively, for 30 min collections lung pericytes were seeded on 96-well plates with 5 × 10^3^ cells per well. The pericytes were seeded in GA medium and allowed to adhere overnight in the appropriate incubator. The following day the pericytes were treated with 4T1 TCM or GA as a control. After 30 min the treatments were removed, the cells were washed once with ice cold PBS, and collected immediately using the cell-2-cDNA kit (AM1723, Invitrogen). Multiplex qPCR was performed with cDNA samples diluted 1:4 and IDT prime time master mix (2x) (1055771, IDT). Primers for house-keeping gene *Rplp0* were used as a concentration of 250 nM each, while primers for *Klf4* were used at 500 nM, probes used at 500 nM. All primers were designed using IDT and sequences can be found in Supplementary Table 2. Analysis was performed using the ∆∆Ct method, using *Rplp0* as the reference gene, and data is presented as average fold change ± standard deviation (SD).

### Morphological analysis

Phase contrast images were collected using the BioTek Lionheart FX automated microscope (LFXW-SN, Agilent). Individual pericytes were manually assigned a morphological subtype based on appearance, according to previously published criteria^44^.

### Immunofluorescence

Millicell EZ 8-Well chamber slides (PEZGS0816, Millipore Sigma) were prepared with 0.06 mg/mL collagen, for a minimum of 2 h at 37 °C. The collagen was removed from the wells and rinsed once with sterile PBS prior to seeding the cells. Pericytes were seeded at 3 × 10^4^ cells per chamber in 150 µL GA medium and allowed to adhere overnight. The next day the medium was removed from the chamber slides and the cells were fixed with ice cold 70% methanol for 7 min. The cells were permeabilised with 0.5% Triton X-100, blocked with 10% donkey serum (S30-M, Millipore Sigma)/3% Fish Gelatin (G7765, Sigma Aldrich) and incubated with primary antibodies at 4 °C overnight. The following primary antibodies were used; rabbit anti-MYH11 (ab224804, Abcam), rabbit anti-PDGFRb (ab32570, Abcam), recombinant Alexa Fluor 647 anti-NG2 (ab283639, Abcam), and mouse anti-ACTA2 FITC conjugated (F3777, Millipore Sigma). The following day the cells were washed with 0.1% Tween20 (655204, Millipore Sigma) before incubation with donkey secondary antibodies (Jackson ImmunoResearch) for 1 h at room temperature, protected from light. The cells were washed again in 0.1% Tween20 solution followed by incubation with DAPI (D1306, Thermo Fisher Scientific) and finally mounted with ProLong™ Gold Antifade mountant (P36930, Thermo Fisher Scientific). Images were obtained on the Nikon SoRa spinning disk microscope. Fluorescence intensity was quantified for each cell using a nuclear mask in ImageJ. Three images per batch per stain were analyzed. Positive cells were determined using secondary only control images and plotted as a percentage of the whole population.

### Tube formation assays

10 µL of ice cold Matrigel (3432-001-01, R&D) was loaded onto 15 µ-angiogenesis slide (81,506, Ibidi) and Matrigel was allowed to polymerise for 30 min at 37 °C. hUVECs were trypsinised, centrifuged and washed in endothelial medium without FBS or growth factors. hUVECs were seeded at 1 × 10^4^ cells per well in 50 µl of serum free medium onto the Matrigel. For pericyte-EC tube formation assays, pericytes stably expressing the zsGreen fluorescent protein were added to the hUVECs at a ratio of 1:5 for brain pericytes or 1:10 for lung, bone and liver pericytes (Pericyte: EC). Tube formation was imaged every 30 min for 8 h using the BioTek Lionheart FX automated microscope (LFXW-SN, Agilent) and analyzed using the Angiogenesis Analyser plugin for ImageJ.

### Statistical analysis

Data are presented as mean ± SD. Where relevant, statistics were performed on the mean values of the independent experiments. Statistical tests were performed using GraphPad Prism 5 software and statistical significance was accepted at the 95% confidence level. Image analysis was performed using ImageJ^72^.

## Supplementary Information

Supplementary Information.

## Data Availability

All data generated or analyzed during this study are included in this article and Supplementary Information files.
